# Towards Scalable and Efficient Architecture for Modeling Trust in IoT Environments

**DOI:** 10.3390/s21092986

**Published:** 2021-04-24

**Authors:** Mustafa Ghaleb, Farag Azzedin

**Affiliations:** Information and Computer Science Department, King Fahd University of Petroleum and Minerals, Dhahran 31261, Saudi Arabia; g200905270@kfupm.edu.sa

**Keywords:** IoT, chord, fog architecture, trust modeling

## Abstract

The *Internet of Services* (IoS) is gaining ground where cloud environments are utilized to create, subscribe, publish, and share services. The fast and significant evolution of IoS is affecting various aspects in people’s life and is enabling a wide spectrum of services and applications ranging from smart e-health, smart homes, to smart surveillance. Building trusted IoT environments is of great importance to achieve the full benefits of IoS. In addition, building trusted IoT environments mitigates unrecoverable and unexpected damages in order to create reliable, efficient, stable, and flexible smart IoS-driven systems. Therefore, ensuring trust will provide the confidence and belief that IoT devices and consequently IoS behave as expected. Before hosting trust models, suitable architecture for Fog computing is needed to provide scalability, fast data access, simple and efficient intra-communication, load balancing, decentralization, and availability. In this article, we propose scalable and efficient Chord-based horizontal architecture. We also show how trust modeling can be mapped to our proposed architecture. Extensive performance evaluation experiments have been conducted to evaluate the performance and the feasibility and also to verify the behavior of our proposed architecture.

## 1. Introduction

With the proliferation of Industry 4.0 and its relation with *Cyber Physical Systems* (CPS) and *Internet of Things* (IoT), a new type of services, *Internet of Services* (IoS), is gaining ground where cloud environments are utilized to create, subscribe, publish, and share services. The fast and significant evolution of IoS is affecting various aspects in people’s life and is enabling a wide spectrum of services and applications ranging from smart e-health, smart homes, to smart surveillance. An IoT system, one of the core pillars for realizing IoS, comprises of loosely coupled interrelated decentralized computing devices. These smart objects, when supported by an “anywhere, anytime, and anything connection” become the basic building blocks of IoT [1,2]. As stated in [3,4,5,6], there are 30 billion interconnected nodes and 75 billion nodes are expected by 2025. Utilizing IoT-connected devices can aid in monitoring vital, confined, restricted, and hazardous places and processes [7,8,9,10,11]. In addition, by analyzing sensor-generated and environmental-based data, a new vision can be drawn to understand past and current events and intelligently predict future events. Before these nodes can be utilized, node collaboration and coordination need to be addressed [12,13,14,15] so tasks, which are beyond the scope of a single node, can be accomplished. Having created smart environments to support decision-making, an IoT vision allows different physical objects to perform tasks such as data generation and collection as well as collaboration and coordination with other objects. These autonomous heterogeneous collections of devices that glue together the physical and cyber worlds come with their own challenges that are prone to affect IoS realization. These challenges can be related to technical or behavior issues. Technical issues, such as the deployment environment conditions and transmission impairment, are investigated and addressed by many researchers [16,17,18]. These technical issues focus on details such as verifying the authenticity of a device and determining the authorizations that the device is entitled to access. Technical issues are solved by security techniques, including encryption, data hiding, digital signatures, authentication protocols, and access control methods. Unfortunately, security techniques say nothing about trustworthiness. For example, a digitally signed certificate does not convey if the issuer is an industrial spy, and a digitally signed code does not convey if the code is written by malicious programmers. Behavior issues deal with a wider notion of device’s *trustworthiness* [19,20] and these issues for IoT devices have just recently started to get attention from the research community [19,20,21,22,23,24,25,26]. Building trusted IoT environments is of great importance to achieve the full benefits of IoS. In addition, building trusted IoT environments mitigates unrecoverable and unexpected damages in order to create reliable, efficient, stable, and flexible smart IoS-driven systems. Therefore, ensuring trust will provide the confidence and belief that IoT devices and consequently IoS behave as expected.

Hosting trust models on thing-level devices is not desirable as most applications require more computational and storage capabilities than those available on commercially resource-constrained objects. Consequently, trust models will be limited in functionality and thus unfeasible to deploy full-fledged trust models. On the other hand, we can host trust models on cloud nodes where computational intensive operations can run. This approach also suffers from various issues [12,27,28,29] related to (a) the high latency between IoT things and cloud nodes, (b) maintaining the connection to remote cloud sites because a failure may decrease IoS reliability, (c) and security concerns as private data is transmitted and processed in remote cloud sites.

As stated in the literature [30,31,32], Fog devices are middle links where operational data is processed and stored by small data centers that are reachable with low latency. These middle links connect devices (things) with cloud nodes. Therefore, Fog devices are promising to host trust models for various reasons. First, these devices provide (to physical-proximity users) cloud-like services from the computational power and storage capacity perspectives. Second, such services are provided with low latency and high bandwidth. As stated in [3,33], IoT devices can reach a Fog site with less than 10 ms, whereas the latency between Fog sites is up to 50 ms. On the other hand, the latency between clients to the cloud is much higher, approximately 200 ms, and this number is unpredictable [3,12,34]. With such characteristics, namely (a) low latency, (b) high bandwidth, (c) high computational power, and (d) high storage capacity, Fog sites can be utilized as hosts for trust models.

### Motivation and Contributions

Before hosting trust models, suitable architecture for Fog computing is needed to provide scalability, fast data access, simple and efficient intra-communication, load balancing, decentralization, and availability. As we write, horizontal Fog architectures have been adopted by many researchers and professional organizations [35,36,37]. In 2018, IEEE adopted OpenFog [38] as the reference architecture for Fog Computing. OpenFog is a horizontal architecture of Fog nodes collaborating with each other to sift, extract, and create intelligence. The Industrial Internet Consortium (IIC) also announced that they had merged the OpenFog consortium under one organization [39]. As a result of this merge, a formal definition of Fog Computing is defined as

*“A system-level horizontal architecture that distributes resources and services of computing, storage, control, and networking anywhere along the continuum from Cloud to Things”.*

Following this universal adoption, we were motivated to propose an architecture for IoT that follows the Fog Computing horizontal widely adopted initiative. Such architecture will be used as an enabler for modeling trust. Our goal is to contribute to Fog-based IoT environments so that scalable and efficient horizontal architecture can be built and utilized for trust modeling. As such, our contributions are as follows:

- We propose a horizontal architecture for Fog environments utilizing the Chord protocol.
- We show how trust modeling can be mapped into our proposed architecture.
- We verify the scalability and efficiency of our proposed architecture.

The rest of this research is organized as follows. Section 2 reviews current Fog-based IoT horizontal architectures and summarizes the key gaps that provide the foundations for our proposed scalable and efficient Fog architecture. Section 3 presents our scalable and efficient Fog-based IoT architecture for modeling trust. Section 4 discusses how trust modeling can be mapped to our architecture proposed in Section 3. Section 4 also illustrates how we envision our trust operations by providing the sequence of the steps a service consumer takes through a trust transaction with a service producer. The performance evaluation experiments for the proposed architecture are outlined in Section 5. Realism and limitations of our proposed architecture are presented in Section 6. Finally, Section 7 concludes the article and envisions future directions.

## 2. Related Work

The goal of this section is to shed light on the horizontal architectures of Fog nodes. These Fog nodes allow interaction and data exchange between edge devices. Moving towards the capabilities of Fog nodes, it is possible to utilize the larger number of Fog nodes in a horizontally cooperative environment such that computational and storage resources at the same Fog layer can be utilized. In this section, we review current Fog-based IoT horizontal architectures and summarize the key gaps that provide the foundations for our proposed scalable and efficient Fog horizontal architecture. For clarity and completeness purposes, we also include vertical Fog architectures.

### 2.1. Vertical Fog Architectures

Vertical Fog architectures are formed of more than one layer. The authors of [40] proposed a vertical Fog computing architecture consisting of three layers. This architecture was proposed to utilize Fog resources and minimize delay to latency-sensitive applications. A Fog four-layer architecture was proposed in [41] as part of an overall IoT solution to enable smart cities. In this solution, authors reserved layer 2 and 3 as Fog layers, where layer 3 consists of high-performance and low-consumption edge nodes. Each computational node in Layer 2, on the other hand, is connected to a group of layer 3 Edge nodes. This architecture aims to respond quickly to control undesirable and dangerous events.

Tree-based Fog Computing (TBFC) architectures have been recently proposed by [42,43,44]. In this architecture, communication between sensors and actuators, in the form of processes and data, is distributed and deployed as a tree structure. Root nodes are formed by cloud servers, whereas leaf nodes are formed by edge nodes. A Fog architecture composed of three-layers was proposed by EdgeX Foundry [45]. Working as a technology-agnostic application enabler, this Fog architecture requires a data communication layer using interprocesses communications. This Fog architecture can also be utilized by more than one device and can run on many edge/fog things such as routers, gateways, hubs, and servers.

The research community is starting to focus on service placement, where services are offloaded from the cloud layer to the Fog layer and thus utilize proximity. Recent surveys [46,47] discussed this problem and explored various proposals to solve it. The authors of [46] introduced a service placement taxonomy to classify proposals based on four dimensions: control plan design (distributed, centralized), placement strategy (offline, online), system dynamicity (static, dynamic), and mobility support. The authors of [48] proposed a hierarchical Fog to serve as an offloading service to relieve task load and extend data storage through accessible Fog resources. To execute services at the edge of the network, a three-layer Fog architecture was proposed in [49]. This article raises the awareness to recognize the suitability of IoT edge computing approaches to better address latency and scalability issues and to improve reactivity through traffic confinement and local control decisions and actuation. Evaluation experiments showed promising performance results when adopting simple but effective strategies for proactive migration. Service provisioning at the edge of the network was also the focus of proposing a hierarchical Fog architecture in [50]. As a solution to latency due to unstable connections and failures, the authors in this article proposed to a solution by utilizing the Fog layer. A hierarchy Fog architecture was formed using edge servers with access points via wireless networks. As such, and with the help of cached services on edge servers, latency is reduced and computation is offloaded from cloud servers. Several evaluation experiments were conducted and results show that the average response time of IoT services is reduced.

A three-layer Fog architecture is proposed in [51] to be used by smart-healthcare infrastructures. The proposed architecture takes a service-oriented methodology to ensure efficiency in terms of process flow and process modeling. The authors of [51] provided a use case as their architectural model validation process.

### 2.2. Horizontal Fog Architectures

Horizontal Fog architectures have been proposed by many researchers and professional organizations [35,36,37,52,53,54,55]. In 2018, IEEE adopted OpenFog [38] as the reference architecture for Fog Computing. OpenFog is a horizontal architecture of Fog nodes collaborating with each other to sift, extract, and create intelligence. IIC also announced that they had merged the OpenFog consortium under one organization [39].

Horizontal Fog architecture is proposed in [56] to reduce the Fog nodes energy consumption. In this architecture, the sensor node sends data to one or more Fog nodes. The Fog nodes are linearly interconnected and can receive data from sensors or other Fogs. The data are processed in each Fog node and then sent to neighbor nodes and cloud servers. A research in the field of horizontal Fog architectures was proposed by [37]. The proposed architecture organizes Fog nodes following a model leveraging the network’s resources to provide improved performance. Performance evaluation experiments show that this architecture reduces bandwidth usage and enables fault tolerance by utilizing local Fog nodes.

To fully utilize Fog nodes and exploit their resources, the authors of [36] proposed a *Peer-to-Peer* interconnecting model where Fog nodes form a horizontal architecture maintaining a set of neighbors based on proximity. This proposed architecture complements unifying all available resources at the edge of the network. However, this work did not provide any information about building a Fog computing environment.

Zhang et al. [53] proposed a cooperative Fog architecture for dealing with *Internet of Vehicles* data in smart city. The Fog layer is a federation of distributed local Fog servers, a coordinated Fog server, and cloud servers. A centralized regional coordinator Fog server is incorporated to assign and manage tasks to different Fog servers. Moreover, a Fog coordinator server requests some information from other Fogs. In a nutshell, this Fog network is controlled and coordinated by a local coordinator server. The architecture supports multi-source data acquisition, and each coordinator acquires and stores the data within their members of IoT networks. After processing the data, the refined information can be disseminated to other Fog nodes.

Naranjo et al. [57] proposed a Container-based virtualized network architecture to support real-time IoT applications. Fog nodes are spatially distributed and possibly interconnected to serve local clusters by providing computing and networking resources. Each Fog node acts as a mini data center, and the Fog node locally processes the delay-sensitive requests while the delay-tolerant tasks are forwarded to the cloud. The data are aggregated in the Fog nodes and then routed to the Cloud server. Once Fog nodes are interconnected, there will be inter-container communication between various Fog nodes. Furthermore, this architecture allows container migration between clusters.

Souza et al. [48] proposed a placement algorithm for combined Fog-to-Cloud architectures. In this architecture, resources are divided into distinct areas, including Fog layers and a distributed control unit (CU). CUs manage the communication between different Fogs as well as the Internet backbone. In this architecture, CUs communicate with each other in the same Fog layer. Moreover, CUs can centralize all required tasks within a specific area or deploy the tasks between different Fogs within the same Fog layer.

Masri et al. [52] proposed a Fog-to-Fog communication algorithm to allow Fog nodes to communicate with each other for processing task requests. The Fog node can execute a task and forward it to the cloud as well as collaborate with other neighboring Fog nodes. The collaboration between neighboring Fogs aims to minimize the delay of submitting the task to the cloud. The data location is centralized in the Fog if the storage space is available; otherwise, it is sent to the cloud.

Nguyen et al. [54] proposed a horizontal Fog-to-Fog structure enabled by Information-Centric Networking where each Fog has a logical link to the cloud server and to other Fog nodes in proximity. The data transfer horizontally between Fog nodes and processes the data in a distributed manner. However, the proposed architecture is not discussed, and also the proximity between neighboring nodes is assumed without any real measurements. Data discovery and routing can be done by the cloud or utilizing name-based routing (NBR). In the case of utilizing NBR, each Fog node maintains routing information such that Fog nodes discover and route named data without support from the cloud.

Naranjo et al. [55] proposed a Fog-based architecture for managing applications of the *Internet of Everything* (IoE) in smart city. The Fog layer consists of multiple Fog nodes that can communicate with each other, utilizing a direct hopping system to process incoming data from the IoE layer and transfer data to other Fog nodes. Each fog node has a local database for storing data of IoT applications. Moreover, the authors proposed a routing algorithm to identify the path for transferring the data between Fog nodes.

**Figure 1:**
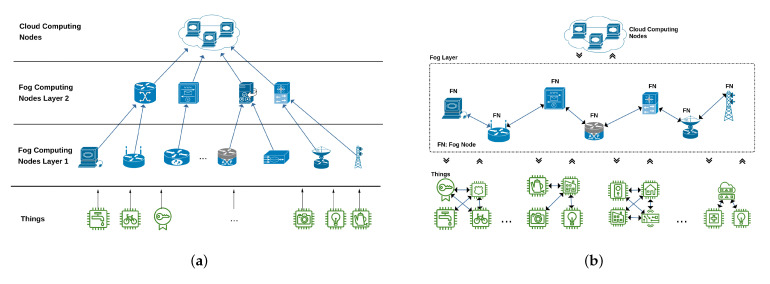
(**a**) Vertical Fog architecture. (**b**) Horizontal Fog architecture.

Figure 1a,b shows the hierarchical and horizontal Fog architectures in general.

### 2.3. Summary

Vertical-based architectures cause overheads and delays as messages need to pass through several layers. More importantly, restructuring can be prohibitively complex, especially where Fog nodes are heavily interconnected due to churn rate and mobility [58,59].

In spite of the universal adaptation of horizontal Fog architectures, issues such as data location, memory storage, and search lookup cost are still key gaps that motivated and provided the foundations for our proposed scalable and efficient Fog architecture. In a cooperative Fog environment, data location solutions are needed where data are not stored in one location but rather distributed across the Fog network. Current architecture solutions at the Fog layer adapt one of the following options. One option is that each node knows the location of all participated nodes in the network. In this case, resolving a lookup is fast O(1), but the routing table is large O(N), where *N* is the number of participated nodes in the network. An alternative option is that each node maintains a pointer to the location of its immediate successor. In this case, the node has small routing table of size O(1), but resolving a lookup would require O(N) in the worst case. In summary, for these two options, if each node knows the location of all participated nodes, then lookups operations have good performance at the expense of larger routing tables. On the other hand, if each node only knows its successor, then routing tables can be small, but the lookup operation takes O(N) messages in the worst case.

Fortunately, Chord [60] is an example of a suitable solution that efficiently resolves lookups while minimizing routing tables. Utilizing Distributed Hash Table (DHT), Chord distributes resources among a set of nodes in the network and locates these resources efficiently. That is, every node knows *m* other nodes in the Chord ring. For example, node with ID 32 would maintain mapping for $32+2^{i-1}$mod$2^{m}$, where *i* points to the successor of $n+2^{i}$. Due to the structure of Chord, these lookups require O(log(N)) hops [60,61].

This motivated us to utilize Chord at the IoT Fog layer and propose an architecture with the following characteristics:

- Enables Fog sites to complement each other in order to satisfy the needs between user’s devices and Cloud Computing centers.
- Each Fog node needs to know the location of O(log(N)) other nodes. Therefore, it scales well with the number of Fog nodes.
- Efficient location solution since lookups take O(log(N)) messages.
- Lookup information is maintained as Fog nodes join and leave the system.

## 3. Proposed Chord-based Fog Architecture

**Figure 2:**
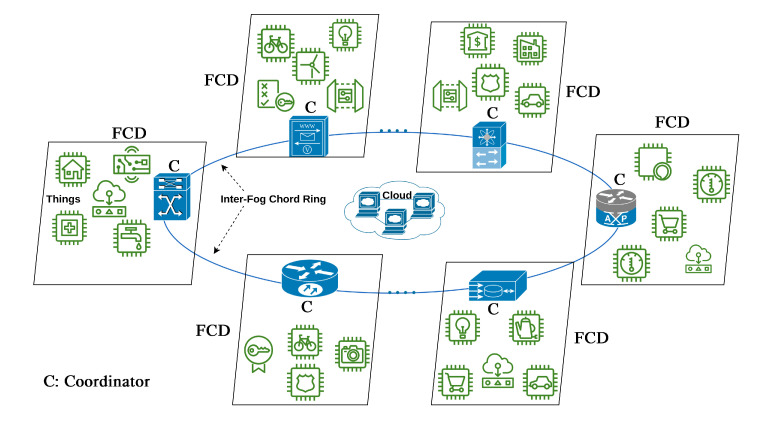
Proposed Chord-based Fog architecture.

In our proposed architecture, shown in Figure 2, we focus on the Fog layer and present a new Fog architecture that minimizes lookup and storage complexity. A straightforward approach to our proposed Chord-based architecture would be to consider each node (Fog-level or thing-level) of the environment as an entity. To reduce the number of nodes, the nodes are aggregated into Fog computing domains (FCDs) as illustrated in the figure. The aggregation, however, has some interesting side effects beyond improving scalability. The aggregation process elects one or more Fog nodes as coordinators for a given FCD. For illustration purposes, Figure 2 shows only one coordinator for each FCD. Because the coordinator is representing the whole FCD, it is held responsible for any violations by the FCD members. The coordinator is expected to manage the member nodes and represent its FCD within the global Fog community. The global Fog community (i.e., Coordinators) is connected with a Chord ring.

For the rest of this section, we present a systematic approach of defining all system components to satisfy the needs and requirements in order to design a coherent and well-running system. Both the functional and the operational architectures of our proposed system are presented to illustrate the working order of the various system components as well as information flow between these components. We describe how operations are employed to accomplish functions. The primary objective is to show the derivation of operational profile from functional profile.

### 3.1. Functional Architecture

**Figure 3:**
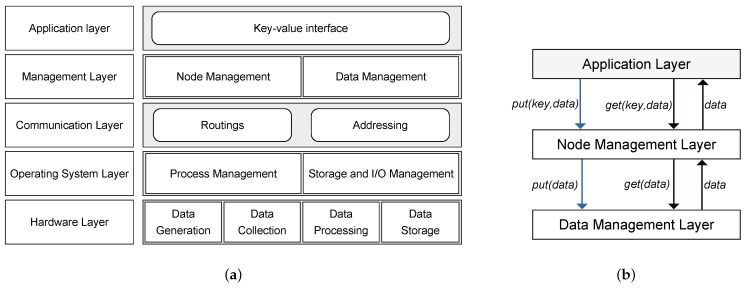
(**a**) Functional architecture of the proposed Chord-based Fog system. (**b**) Logical structure between application and management layers.

Figure 3a shows the functional architecture of the proposed Chord-based Fog architecture. The aim of this figure is to show the segregation of functionalities across the different layers of the architecture. The hardware layer contains the IoT basic building blocks that are responsible for collecting and storing data. This layer encompasses devices ranging from low-end devices such as sensors and actuators to high-end devices such as cloud servers. IoT operating systems such as Contiki and TinyOS manage hardware devices, processes running on these devices, and I/O operations. Connecting these devices needs to be established through the communication layer in a securely, efficiently, and cost-effectively way. Functions at this layer include routing, addressing, and forwarding that need to be carefully designed and deployed because of challenges concerning the resource-constrained nature.

In IoT systems, data sources (devices) at the things layer generate data items, and thus a management layer is vital to orchestrate both nodes and data and must answer the following big challenges:

- Where shall a data item be stored.
- How does a requester find the actual location of a data item.
- How to limit the complexity for communication and storage.
- How to ensure robustness and resilience in case of faults and frequent changes.

Figure 3b shows the logical structure between the application layer and the management layer. The application layer accesses the node management layer using a simple interface represented as a key-value tuple to manipulate IoT data. One of the objectives of this interface is to hide the actual lower layers’ implementation complexity. The node management layer deals with all issues related to managing a dynamic Fog node environment. The implementation of this layer provides the logic for managing the Fog nodes. Fog nodes are organized in a structure that is maintained and adapts dynamically as Fog nodes join, fail, or leave the system. On the other hand, the data management layer uses Fog nodes as hash table buckets, where each Fog node stores only a fraction of the generated data. Data failure is also handled by the data management layer.

### 3.2. Operational Architecture

In the operational profile, we include details such as tasks, operational elements, and information flows required to accomplish or support the functionalities of our proposed architecture. We show the operational architecture for both management components, namely, node and data management.

#### 3.2.1. Node Management

Node management deals with tasks related to node discovery and the dynamic joining/leaving of Fog nodes. As such, node management is concerned with node positioning to ensure (a) robustness and resilience in case of faults and frequent changes, and (b) minimizing communication complexity between Fog nodes. As shown in Figure 2, Fog nodes are structured in peer-to-peer manner. This design will be self-managed, thus eliminating the need for centralized control over the system.

**Figure 4:**
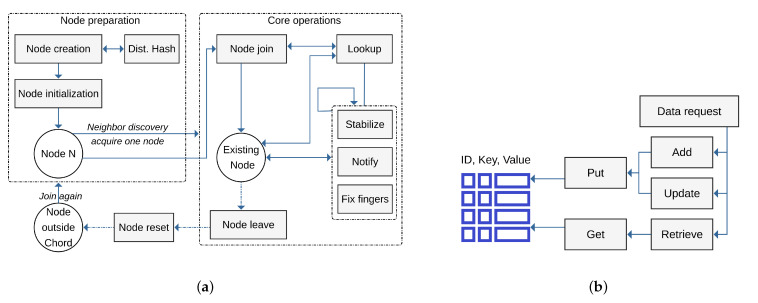
(**a**) Node management operational architecture. (**b**) Data management operational architecture.

As shown in Figure 4a, Fog nodes need to join, but first they have to be created by *node creation* operation that calls the *distributed hash* function to assign a new Id to the node by hashing its IP address. Then, the node uses the *node initialization* operation to initialize its finger table, successor, and predecessor lists. After broadcasting messages to discover and pick one neighbor, the node can now manipulate the Chord ring through two interfaces, namely, *node join* and *node leave*. Once the node is part of a Chord ring, we refer to the node as *existing node*. If the node leaves the Chord network, it resets its successor, predecessor, and finger table entries and becomes outside the network and can rejoin. The Chord resolves a query through a *lookup* operation. In addition, there are three more operations, namely, *stabilize, notify,* and *fix fingers.* These three operations are used to maintain good lookup performance despite continuous failure and joining of nodes.

#### 3.2.2. Data Management

The data management component utilizes the structure established by the node management component to deal with tasks related to data allocation and provisioning as well as maintaining data pools in a distributed yet efficient manner. Through the operations **put** and **get**, as shown in Figure 4b, data items can be propagated in the Chord ring. At every Fog node, the DHT strategy is applied to define where the data item will be stored. To handle the DHT, Chord is utilized such that every Fog node will be responsible for keeping only O(log(N)) addresses of other Fogs.

## 4. Trust Model Mapping

### 4.1. Overview

Our trust model is mapped into the proposed architecture illustrated in Figure 2 by representing and using two trust relationships: inter-Fog and intra-Fog. Coordinators are connected through Chord and form inter-Fog Chord ring whereas things are members in their respective domain referred to as intra-Fog domain managed by a coordinator.

### 4.2. Inter-Fog Trust Representation and Usage

The trust model maintains trust levels between coordinators of FCDs. That is, each coordinator has a global trustworthiness in the eyes of other coordinators. This global reputation of a coordinator is affected by how trustworthy its members (resources and clients) behave when engaged in a transaction. These member nodes collectively contribute to the trustworthiness of their coordinator. The trust levels that a coordinator believes of other coordinators directly interacted with are kept with the coordinator as past experience. For a transaction between two coordinators, the actual interaction occurs at the member nodes level (i.e., between resources or clients).

Consider FCD formed by *n* nodes. Suppose a new node is interested in joining the FCD, it would negotiate with the FCD’s coordinator regarding the trust level that would be bestowed upon it. Ideally, the joining node would like to be trusted at the highest possible level and may lay claim based on references (i.e., other FCDs) from prior associations. The coordinator has conflicting requirements: (a) it wants to present its nodes as highly trustworthy to the outside FCDs because the value of the highly trustworthy nodes will be much higher than the nodes that are less trustworthy, and (b) it does not want to overestimate the trustworthiness of a node because its own reputation will suffer if the node turns out to be less trustworthy. This conflict drives the coordinator to make choices that are as close to optimal as possible.

### 4.3. Intra-Fog Trust Representation and Usage

Each coordinator manages its member nodes (clients or resources) and deploys mechanisms to allow them to join, operate, and leave the pool of clients or resources. This management needs to be done because the behavior of the nodes affect the FCD’s reputation and hence its interaction with other FCDs. As nodes join or leave a FCD, the FCD’s coordinator needs to manage these operations accordingly.

Suppose that a node wants to join FCD, the node negotiates with the FCD’s coordinator who can either reject or accept the join request. The node may lay the join request based on references from prior associations. References are given by other coordinators and hence can affect the referee coordinator’s honesty if the node’s behavior is not up to the reference. As a consequence, the referee coordinator might be isolated and no more references are accepted from it. This also has a consequence on the referee coordinator to be isolated from coordinators’ references set.

Upon joining, nodes are clustered in accordance with their behavior. If the node has no references, it will be placed into the lowest cluster. Based on its references, a node will be placed in an appropriate cluster matching the node’s trustworthiness. The coordinator is responsible for promoting or demoting nodes among the clusters based on the nodes’ behavior. The coordinator maintains an *internal trust table* (ITT) that includes trust levels for its member nodes. Each time a node participates in a transaction, the coordinator adjusts the node’s trust level in the ITT by getting reviews from other coordinators participated in the transaction.

### 4.4. Trust Transaction Example

**Figure 5:**
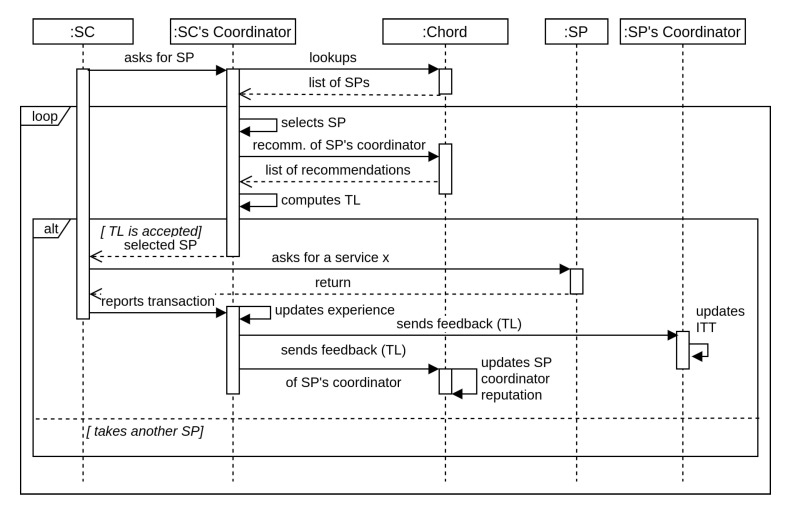
Sequence of steps during a trust transaction.

In this section, we illustrate how our trust model operates by providing the sequence of the steps a Service Consumer (SC) takes through a trust transaction with a Service Provider (SP). Here, the SC and SP are things-level nodes that are members in FCDs. The sequence diagram in illustrated in Figure 5.

The figure shows an example of SC wanting to engage in a transaction with SP. In such a transaction, there is a trust concern from the SC as well as the SP. Let us assume that SC is concerned about SP’s trustworthiness and hesitant to consume its service. To make a decision about whether to have a transaction with SP, our trust model enables the SC through its coordinator to rely on SP’s reputation as well as past experience with SP.

First, the SC contacts its coordinator asking for an SP that provides a specific service. The coordinator utilizing the inter-Fog Chord ring, sends a lookup query, and gets a list of all SPs. The coordinator selects a trustworthy SP by consulting its experience table. The coordinator also collects recommendations from other coordinators regarding the SP coordinator. Next, the SC coordinator receives the recommendations in the form of opinions as explained in Section 4.5. These recommendations are weighted and combined to get the final indirect trust towards the SP’s coordinator by applying discounting and consensus operators together. Finally, the coordinator computes the trust level (TL) based on the SP coordinator’s reputation and the coordinator’s experience with SP coordinator. If the computed TL is satisfactory, the SC engages in the transaction with the SP.

After the transaction, the SC coordinator needs to disseminate its experience with the SP coordinator. This information is disseminated locally and globally as follows:

- The SC coordinator updates its experience as described in Section 4.5.
- The SC coordinator also sends this experience to the SP coordinator so that the trustworthiness of SP is updated (i.e., updating the ITT).
- The SC coordinator disseminates this experience through Chord so that the reputation of SP coordinator is updated. This information is basically aggregated with previously stored reputation using consensus operator as described in Section 4.5.

### 4.5. Evolving Trust Using Subjective Logic

Subjective logic, which is a particular form of belief theory, builds on the belief that trust is subjective and everyone differently experiences it [62,63]. It is not feasible for an evaluator node to identify all related trust metrics to assess another node’s trust value, and this will lead to the fact that the trust is calculated with insufficient evidence [62,63]. The subjective logic is suggested to be the most appropriate for modeling trust in the fog computing environment [64,65] as each node computes another node’s trust value it encounters subjectively. Subjective logic utilizes opinions as input and output variables. An opinion from the work in [63] is defined as

(1) $$\omega_{x}=\left(b_{x},d_{x},u_{x},a_{x}\right)$$

where $b_{x}$ indicates the amount of belief that the node *x* is trustable, $d_{x}$ refers to the disbelief, $u_{x}$ refers to the uncertainty of the trust relation, while the atomicity $a_{x}$, the base rate of *x*, is the prior probability of *x* without any evidence. The sum of $b_{x},d_{x}$ and $u_{x}=1$. Usually, the atomicity $a_{x}$ is equal to 0.5 to give the opinion an equal probability of giving true or false output.

In order to get the above subjective trust tuple values, the node computes them from the obtained observations, reported experiences from SCs, as illustrated by the following Equations (2)–(4):

(2) $$\begin{array}{cc} b_{x}= & \frac{pos}{pos+neg+k} \end{array}$$

(3) $$\begin{array}{cc} d_{x}= & \frac{neg}{pos+neg+k} \end{array}$$

(4) $$\begin{array}{cc} u_{x}= & \frac{k}{pos+neg+k} \end{array}$$

where pos and neg are positive and negative observations as evaluated by a SC towards a SP, respectively, while k=1.

The single trust value can be extracted from the opinion using this Equation (5). It is called the opinion’s probability expectation value $E_{x}$.

(5) $$E_{x}=b_{x}+\left(a_{x}\times u_{x}\right)$$

Note that the $a_{x}$ determines how uncertainty shall contribute to $E_{x}$.

The discount operator ⊗ is utilized to scale the recommendations the node received with the previous opinion the node has about the recommender. For instance, when Fog *i* wants to compute the indirect trust values regarding service provider *j* utilizing recommendation from the intermediate Fog *k*, the discount operator is utilized. In this case, the weight of trusted recommenders will result in high trust values, and vise versa. Suppose a Fog *i* has an opinion of a recommender Fog *k* as $T_{i,k}=\left(b_{i,k},d_{i,k},u_{i,k},a_{i,k}\right)$ and the recommender Fog *k* has an opinion of the service provider *j* as $T_{k,j}=\left(b_{k,j},d_{k,j},u_{k,j},a_{k,j}\right)$. Then, the indirect trust values of *j* as assessed by *i* based on recommendation from *k* is computed as

(6) $$\begin{array}{c} T_{i,j}=\left(\begin{array}{c} b_{i,k}b_{k,j}\\ b_{i,k}d_{k,j}\\ d_{i,k}+u_{i,k}+b_{i,k}u_{k,j}\\ a_{k,j} \end{array}\right) \end{array}$$

The consensus operator ⊕ is utilized to average two opinions together. Suppose Fog *i* and *k* have recommendations about service provider *j* as $T_{i,j}=\left(b_{i,j},d_{i,j},u_{i,j},a_{i,j}\right)$ and $T_{k,j}=\left(b_{k,j},d_{k,j},u_{k,j},a_{k,j}\right)$, respectively. The combined recommendations for *j* is calculated as

(7) $$\begin{array}{c} T_{i,j}=\left(\begin{array}{c} \frac{b_{i,j}\times u_{k,j}+b_{k,j}\times u_{i,j}}{den}\\ \frac{d_{i,j}\times u_{k,j}+d_{k,j}\times u_{i,j}}{den}\\ \frac{u_{i,j}\times u_{k,j}}{den}\\ \frac{a_{i,j}\times u_{k,j}+a_{k,j}\times u_{i,j}-\left(a_{i,j}+a_{k,j}\right)\times u_{i,j}\times u_{k,j}}{den_{2}} \end{array}\right) \end{array}$$

where $den=u_{i,j}+u_{k,j}-u_{i,j}\times u_{k,j}$ and $den_{2}=u_{i,j}+u_{k,j}-2u_{i,j}\times u_{k,j}$.

Overall indirect trust can be obtained by applying discounting and consensus operators together on the received recommendations as subjective trust [65]. Suppose a Fog node has a list of recommenders and has trust values about them at time *t* as $T_{i,r1}^{t},T_{i,r2}^{t},\ldots ,T_{i,rn}^{t}$ and these recommenders have trust values about service provider *j* at the time *t* as $T_{r1,j}^{t},T_{r2,j}^{t},\ldots ,T_{rn,j}^{t}$, then the final indirect trust of *j* after applying the discounting and consensus operators is computed as

(8) $$\begin{array}{cc} T_{i,j}^{t}\left(t\right)= & \left(T_{i,r1}^{t}⊗T_{r1,j}^{t}\right)⊕\left(T_{i,r2}^{t}⊗T_{r2,j}^{t}\right)\\ & ⊕\ldots ⊕(T_{i,rn}^{t}⊗T_{rn,j}^{t}) \end{array}$$

## 5. Performance Evaluation

In this section, we show our evaluation of the proposed Chord-based Fog architecture. We set up the experimental environment considering specific configurations that are required by the architecture. Then, we run a set of experiments, collect the results, and perform the analysis. Section 5.1 describes the details of building the experimental platform, while Section 5.2 outlines the approach followed in conducting the experiments. Section 5.3 verifies the correctness of our implementation by comparing the obtained results with results presented in the original Chord paper [60]. Section 5.4 discusses the effect of mobility on the performance of our proposed Chord-based Fog architecture. Section 5.5 discusses the energy consumption after one hour of running Chord-based network utilizing NullRDC and ContikiMAC protocols.

### 5.1. Experimental Environment

Here, we present and discuss the test environment components to evaluate our proposed architecture. Various IoT simulators have surfaced in recent years, yet Contiki’s [66] popularity and wide acceptance as IoT operating system made us use this open source operating system with its built-in Cooja simulator [67,68,69]. We first summarize the configurations setup and their values used throughout our experiments in Table 1 and then give a brief overview behind using Contiki and Cooja.

#### 5.1.1. Contiki Operating System

Contiki OS is flexible, lightweight, multi-tasking, and highly portable operating system for IoT. It is a powerful toolbox for structuring sophisticated wireless systems. Contiki comes with a rich set of features and some of them can be outlined as follows:

- Supporting various platforms such as Z1, Wismote, MicaZ, and SKY motes; microcontrollers such as Atmel AVR family, TI MSP430 family, and ST STM32w; and radios such as the Texas Instruments CC1020, CC2420, and CC2520, and RFM TR1001.
- Supporting dynamic and efficient memory management and runs utilizing small memory sizes, i.e., RAM (10 Kbytes) and ROM (30 Kbytes).
- Providing two kinds of networking: non-IP networking and IP networking. The former is enabled through Rime stack and the latter is enabled through uIPv4 and uIPv6 stacks.
- Implementing the adaptation layer, 6LoWPAN, to support uIPv6 seamless operations.
- Providing various implementations of RDC and MAC protocols.
- Comes with Cooja, a built-in flexible hardware-level emulator.

**Figure 6:**
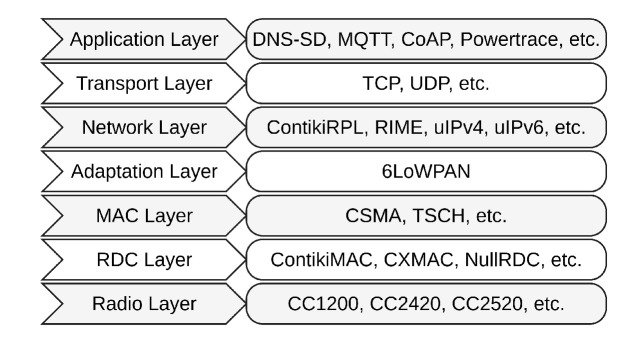
IoT-enabling protocols implemented in Contiki OS.

Figure 6 shows some of the IoT-enabling protocols that implemented on Contiki OS.

#### 5.1.2. Cooja Simulator

Cooja is a cross-layer simulator developed for IoT nodes running Contiki OS. IoT nodes can be simulated in Cooja either at the network level, OS level, or machine code instruction set level. At the network level, Cooja simulates IoT nodes by implementing them in Java to simulate abstract and high-level operations such as sending/receiving packets. While at the OS level, the nodes’ code is Contiki C running on the host machine natively without considering hardware constraints of sensor nodes. Unlike at the machine code instruction set level, the IoT nodes are emulated using MSPSim and the loaded codes are natively a Contiki C code compiled to the target hardware device. We can summarize the core features of Cooja as follows:

- It mimics the same instruction sets of IoT nodes and this facilitates modeling of the fine-grained node behavior.
- It runs a simulation at the hardware-level utilizing genuine hardware profiles. As a result, the compiled code can be uploaded into real platform.
- It evaluates the proposed solutions under real conditions by exploiting the available network models such as topology, radio propagation, medium interference, and link quality models.
- It eases the development and evaluation of IoT network protocols and supports both command line and graphical interfaces.

Although Cooja was designed for WSN simulation, not for Fog computing networks, the support of emulation of various platforms and IoT protocols with mobility support making it a good environment to carry out our intended study. The alternative simulators such as iFogSim lack necessary functionalities to address our proposed solution. Some of these functionalities are churn rate and mobility [70]. Furthermore, many researches simulated their Fog-related work using Cooja [71,72,73,74,75,76,77].

### 5.2. Experimental Methodology

For a deployed architecture, we run a set of experiments that follow the same methodology to ensure their valid comparability.

**Figure 7:**
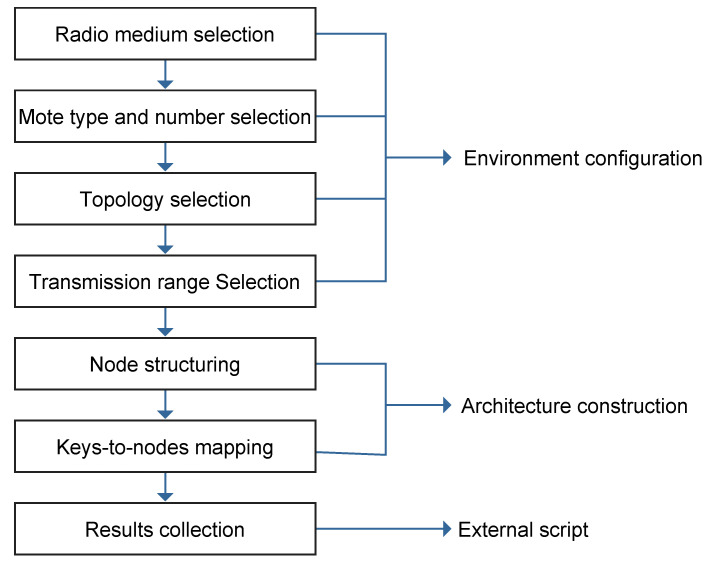
Experimental setup approach.

Figure 7 depicts the phases while performing each individual experiment. The phases of running experiments start by utilizing Cooja simulator to

- select a radio medium model such as distance/constant loss Unit Disk Graph Model (UDGM), Directed Graph Radio Medium (DGRM), or Multi-path Ray-tracer Medium (MRM);
- select a mote type from the supported motes such as Z1, Wismote, MicaZ, or SKY mote and select the number of motes;
- select a network topology such as uniformed 2D-Grid or random positioning; and
- select the transmission range for the populated nodes.

Then, the Chord architecture utilizes Contiki and Cooja to construct the Chord network and execute mapping procedures to insert/retrieve key-value pair into/from hash tables. After selecting the performance evaluation metrics, Cooja simulation scripts and other tools such as Gnuplot were used to collect, save, analyze, and display simulation results.

### 5.3. Verification of the Proposed Model

In this section, the proposed architecture is evaluated. As the proposed architecture is a Chord-based solution, its correctness is verified by comparing the results to the corresponding Chord-related results presented in the original paper [60]. The conducted comparison considers a wide range of metrics such as the key load balancing, path length, miss rate under simultaneous node failures, churn rate, and lookup latency. The following subsections discuss these in detail.

#### 5.3.1. Load Balancing

**Figure 8:**
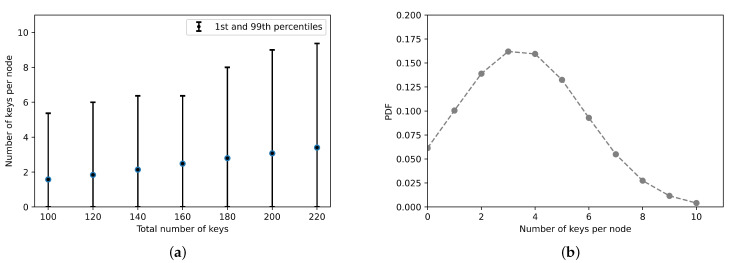
(**a**) The 1st and 99th percentiles and mean values of number of stored keys by a node in a network with 64 nodes. (**b**) The probability density function (*PDF*) of number of keys per node. The total keys is 220.

In this section, the Chord performance is investigated in terms of its ability in allocating the keys to Fog nodes evenly. The conducted experiments consider distributing various number of keys in each experiment, ranging from 100 to 220 keys in step of 20 keys. A number of 64 Fog nodes is considered in all experiments. Let *N* and *K* refer to the number of Fog nodes and keys, respectively. The typical number of keys per Fog node would be K/N [60]. Figure 8a depicts the 1st and the 99th percentiles and mean values of the number of keys per node. As shown in the figure, the number of keys per node increases linearly with the number of the allocated keys. The figure also shows that, in all cases, some nodes do not store any keys. This is attributed to the fact that the nodes IDs do not cover the whole identifier space uniformly [60]. To scrutinize the load balancing further, Figure 8b presents the probability density function (PDF) of the number of keys per node. For the sake of simplicity, the figure only shows results when the number of keys and nodes are 220 and 64, respectively. From the figure, the maximum number of allocated keys per node is 10. For comparison, the 99th percentile is 2.75×y, where *y* is the mean value.

#### 5.3.2. Path Length

Evaluating any routing protocol in terms of the path length between nodes is utmost of importance. The path length refers to the number of nodes traversed to resolve a query as defined by Chord protocol [60]. The Chord protocol specifies that with high probability, the typical path length is O(log(N)).

**Figure 9:**
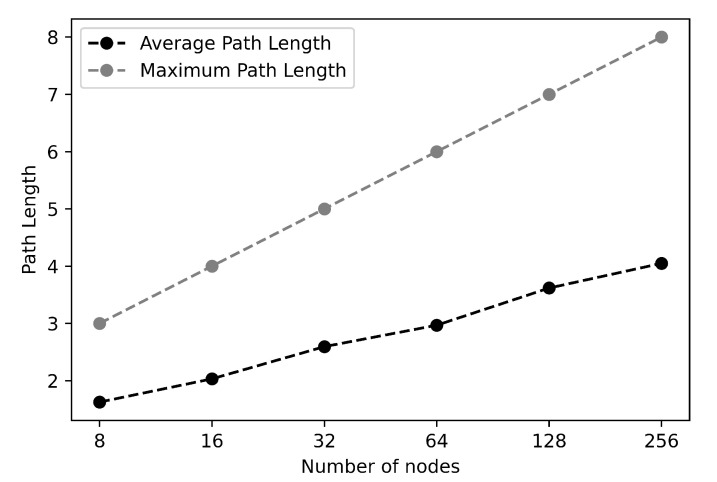
Average and maximum path length as a function of the network size.

The considered network size is $N=2^{k}$ and the *k* varies from 3 to 8 and run experiments for each size. Each node generates a random set of keys from a pre-deployed keys to query the system. Figure 9 shows the maximum and the average path length as a function of the network size. As shown in the figure, the maximum path length is O(log(N)) for all considered network sizes. Furthermore, the average path length obtained from the experiments is almost the same as that suggested by Chord protocol, 1/2×log(N).

**Figure 10:**
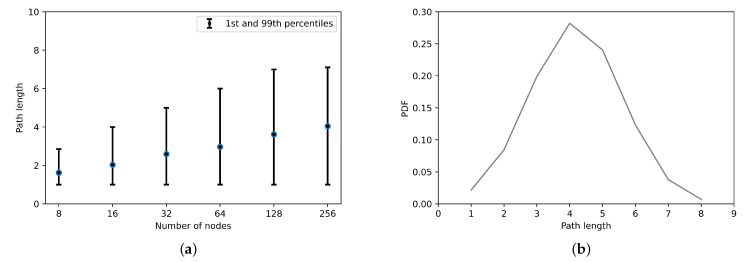
(**a**) The path length as a function of network size. (**b**) The *PDF* of the path length in the case of a $2^{8}$ node network.

For further analysis, Figure 10a presents the means and 1st and 99th percentiles values of the path length. From the figure, one can see that the path length increases logarithmically as specified by the Chord protocol with the number of nodes in the network. Finally, Figure 10b shows the PDF of the path length when the network size is $2^{8}$ nodes. To sum up, the maximum path length when the number of nodes are $2^{8}$ has never exceeded 8 hops in our simulation.

#### 5.3.3. Simultaneous Node Failures

**Figure 11:**
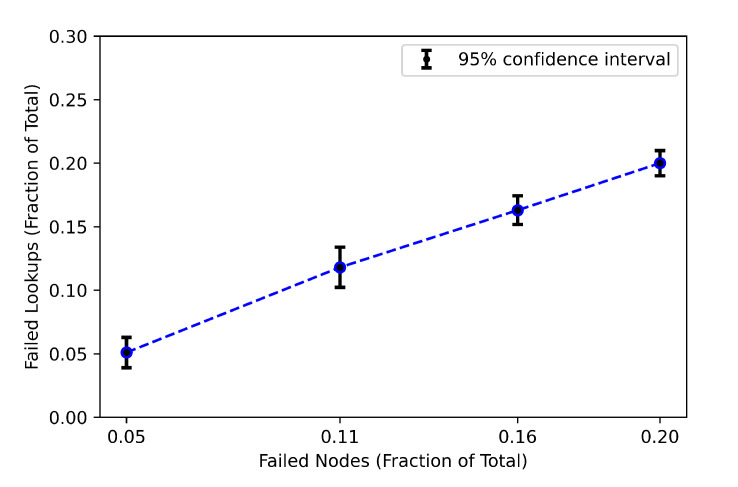
The fraction of lookups that fail as a function of the fraction of nodes that fail.

One of the characteristics of the Chord protocol is the ability to regain consistency in the case of simultaneous nodes failure [60]. Therefore, in this experiment, we investigate how the Chord network remains connected even when a large number of nodes fail simultaneously. The considered network size is $2^{6}$ nodes that stores $2^{8}$ keys and we randomly select a fraction of nodes *p* that fail. We select the percentage of node failures to be p={0.05,0.11,0.16,0.2}. First, we run the experiment until the Chord network is stable and store keys in the corresponding nodes. Then, the nodes failure occur and we wait until the network reaches a steady state after running the stabilization operation and then measure the miss rate which is the fraction of keys that could not be retrieved correctly. We consider the lookup as failed if we do not find the node that was initially responsible for the key we are looking for. We consider an application that stores keys in the corresponding nodes without replication and no recovery of these values after node failures. Figure 11 presents the mean miss rate (lookup failure rate) and the 95% confidence interval as a function of the percentage of node failures *p*. As can be seen from Figure 11, the failed lookups increase linearly with the failed nodes. For example, when the fraction of failed nodes is 0.16% of the total nodes, the mean failed lookups are almost the same as the percentage of failed nodes. We can conclude that there are no significant lookup failures in the Chord network and the result adheres to the assumption of the Chord protocol.

#### 5.3.4. Lookups during Stabilization

**Figure 12:**
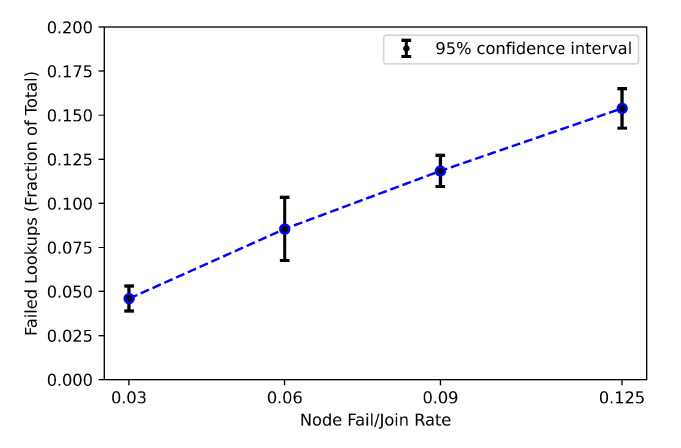
Failed lookups as a function of node fail/join rate.

The impact of churn rate on lookups is investigated in this experiment. The churn rate is defined as the rate at which nodes join or leave the network per time unit [78]. Key lookups may fail and predecessor pointers may be inconsistent due to concurrent joins and leaves. To resolve these inconsistencies, Chord runs the stabilization protocol frequently. Therefore, Chord’s performance is sensitive to the frequency of churn rate versus the frequency of the stabilization protocol invocation. In our implementation, key lookups are generated randomly at a rate of 1 per minute and all nodes send random keys lookups. We have 64 nodes each generating 1 lookup every 1 min, and as a total, we have around 1 lookup per second. Nodes joining and leaving are modeled randomly with an average of 400 s. Each node invokes the stabilization protocol at randomized intervals in average of 60 s. Figure 12 presents the mean miss rate of lookups and 95% confidence intervals as a function of the rate at which nodes leave and join. A node failure rate relates to the fraction of total nodes leaving and joining every 400 s on average. The results shown in Figure 12 are the average of running the experiments approximately two hours of simulated time and each node sends around 50 key lookups. The 95% confidence intervals are calculated based on 10 independent runs.

A failure of lookup is counted if its finger path points to a failed node. As outlined in [60], the probability that one of the failed nodes *k* is in the finger path is around (Average_path_length×k/N). In our case, the probability will be higher since we considered the failure scenarios mentioned in [60] plus failures when the lookup’s packet is lost due to packet loss from different sources such as wireless channel loss and buffer overflow [79]. Therefore, the failed lookups shown in Figure 12 is further affected as a consequence of these losses and is higher than the results shown in [60].

**Figure 13:**
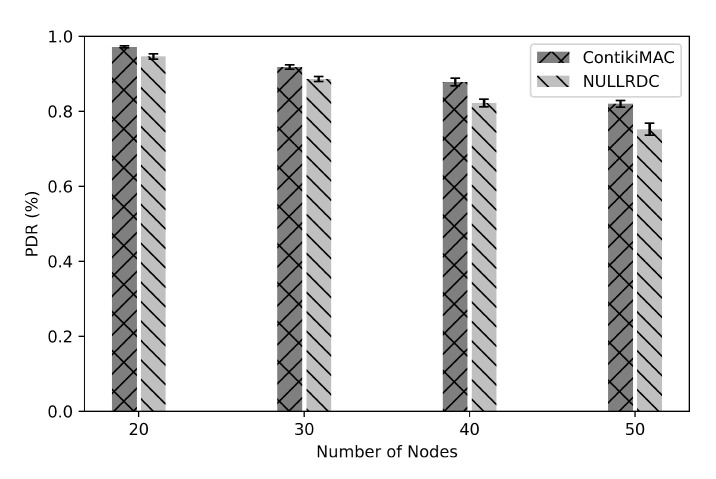
Lookups Packet Delivery Ratio (PDR) as a function of network size.

For better intuition, we run some experiments to measure lookups Packet Delivery Ratio (PDR). Lookups PDR is the fraction of lookup packets successfully received over those sent in lookups operation. Figure 13 shows the Chord network performance in terms of lookups PDR, and this figure presents the 95% confidence intervals and lookups PDR using nullRDC and contikiMAC protocols. In general, and as shown in the figure, the lookups PDR slightly decreases as the number of nodes increases. Note that the lookup success is significantly affected by the routing protocol of the network layer (i.e., RPL in our experiments). In a sense, the lookup message might be dropped due to not being forwarded over the correct path towards the intended destination [80]. As illustrated in Figure 13, ContikiMac obtains a slightly higher lookups PDR compared to NullRDC because NullRDC does not provide any retransmission [81].

As shown in Figure 13, when the number of nodes in the network is 50 nodes, the mean PDR in the case of using NullRDC is 75% and the packets’ loss is 25%. This percentage of loss is added to the percentages of failed lookups during the churn rate experiment, and as a result, it increases the percentage of failed lookups as shown in Figure 12.

#### 5.3.5. Lookup Latency

**Figure 14:**
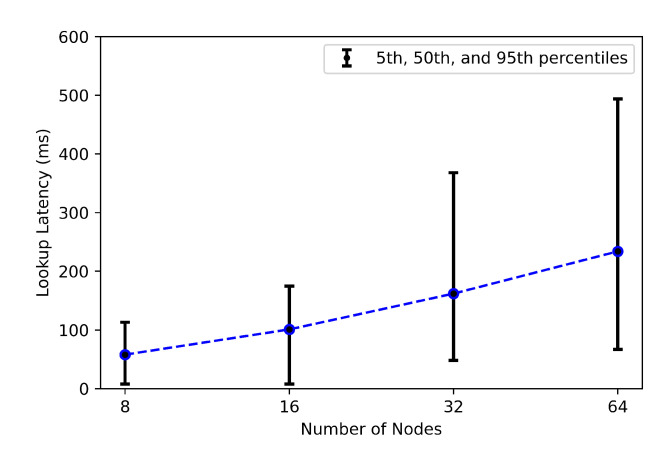
Lookup latency as a function of the total number of nodes.

In this experiment, we measure the latency for sending a query until we get the right location of the node responsible for the desired key. Figure 14 presents the measured latency (5th, 50th, and 95th percentile) and 95% confidence intervals of key lookups over various network sizes ranging from 8 to 64 nodes. Depending on the network size, the 50th percentile latencies range from 58 to 230 ms. As explained in [60], the objective of this experiment is to confirm and demonstrate Chord’s scalability. As the number of nodes increases, the lookup latency grows slowly, and this reinforces the scalability factor of the Chord protocol.

### 5.4. Mobility Effect on Packets Delivery

In order to investigate how the Chord network in IoT will behave when there are some mobile nodes in the system, we run some experiments to measure the PDR of sending key lookups packets. In this experiment, we utilized NullRDC in the RDC layer and network size is 32 nodes with a Chord space of 128. To test the mobility in our system, we utilize two well-known mobility models: “Random Way Point” and “Gauss Markov” by using the BonnMotion tool [82] to generate nodes’ positions. Through the Cooja simulator, we enabled the Mobility plugin to allow the nodes to move during run time. When the mobile node moves, it will probably disconnect from its assigned parent. In order to preserve the connectivity to the RPL Directed Acyclic Graph (DAG), it should discover new available parents in its neighborhood by sending DAG Information Solicitation messages.

**Figure 15:**
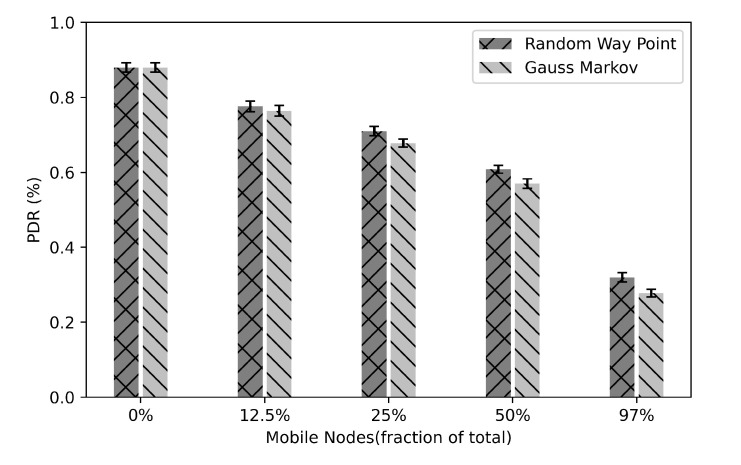
Lookups PDR as a function of the fraction of mobile nodes utilizing Random Way Point and Gauss Markov mobility models.

Figure 15 presents the percentage of lookups PDR as a function of the fraction of mobile nodes using Random Way Point and Gauss Markov mobility models. We start the experiment by making all nodes static. Then, we make 12.5% of nodes mobile and then increase the percentage of mobile nodes to 25%, 50%, and 97%.

The results show that the performance of the system with respect to packets delivery is decreased with the increase of mobile nodes. This degraded performance is due to the changing over time of the positions of nodes that caused loss connectivity of their current parents and hence caused packets to be dropped.

### 5.5. Energy Consumption

This section presents the energy calculation methodology and then discusses the energy consumption obtained from the experiments.

In order to calculate the energy consumption, Energest [83] is utilized. Energest is an energy estimation software module developed for Contiki. It provides a real-time estimation of the consumed energy of individual nodes in different states. To do so, the Energest module tracks the time interval in which the micro-controller stays in *on* state named $CPU_{on}$ or low-power mode state named $CPU_{lpm}$. Furthermore, it tracks the time interval in which the radio transceiver is *on* in the transmission state named $T_{x}$ or the listening state named $R_{x}$. For each component, the energy consumption $E_{s}$ is calculated utilizing Equation (9) presented in [84].

(9) $$E_{S}=\left(T_{S}*C_{S}*V\right)/\left(L\right)$$

where $T_{S}$, $C_{S}$, and *V* are the spent time represented in clock ticks and the current consumed in indicated state *S* and battery voltage *V* respectively, while *L* is the number of clock ticks per second which is equal to 32,768 ticks/s. In our experiment, the Wismote platform is used and as specified in [85] the current requirements for states $CPU_{on}$, $CPU_{lpm}$, $R_{x}$, and $T_{x}$ are 2.2 mA, 1.69μA, 18.5 mA, and 33.6 mA. In this experiment, we collect the results after one hour of running the Chord network with a size of 64 nodes and Chord space of 128 identifiers.

**Figure 16:**
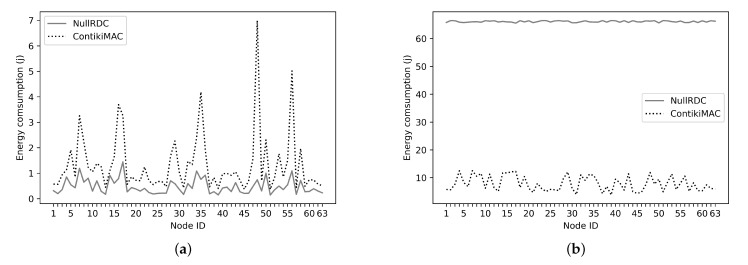
(**a**) Total Consumed energy at $CPU_{on}$, $CPU_{lpm}$, and $T_{x}$ modes. (**b**) Consumed energy at $R_{x}$ mode.

Because the radio transceiver in NullRDC is always on, the majority of energy is consumed during packet reception or idle listening while other related components contributing insignificantly as shown in Figure 16a,b. Note that the $R_{x}$ is the most greedy in terms of energy consumption as shown in Figure 16b.

**Figure 17:**
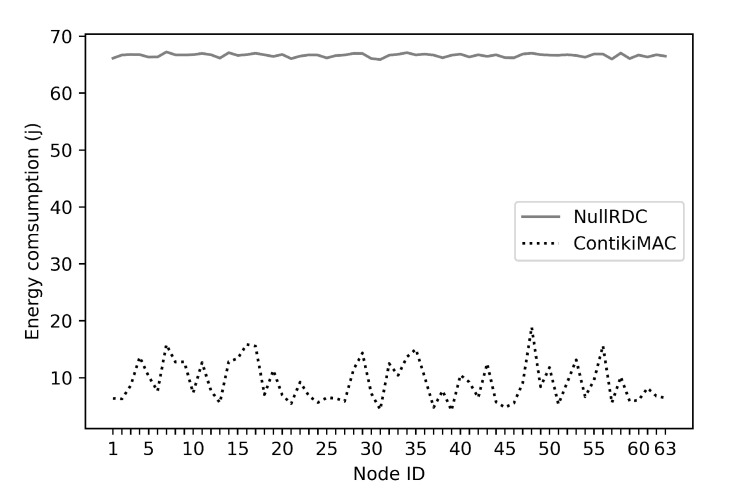
Consumed energy at $CPU_{on}$, $CPU_{lpm}$, $R_{x}$, $T_{x}$, and Rx modes.

To analyze the energy consumption further, Figure 17 shows the per node total energy consumption. The plotted results include $CPU_{on}$, $CPU_{lpm}$, $R_{x}$, and $T_{x}$ modes. It can be seen from the figure that the energy usage using NullRDC is approximately 66.6 Joules on average which is almost 7.2 times higher than ContikiMAC.

Note that Wismote is powered by batteries with a 2xAA (3.0 V) [86]. The nominal charge capacity of these batteries is approximately 1300–3000 mAh. The nominal charge capacity is assumed to be 2500 mAh. To convert mille Ampere Hour (mAh) into Joules, the unit conversion factor is 3600 [87]. Therefore, the energy per Wismote can be calculated as follows: Energy = (nominal charge capacity ×$10^{-3}$× Voltage × Time) Joules. Thus, the Energy=2.5×3×3600=27,000 Joules. Utilizing ContikiMAC as an underline RDC protocol to simulate 64 nodes, the average consumed energy is 9.2 Joules per hour. Therefore, the network will stay functioning 122.3 days on average. This indicates even if the Fog node is a 6LoWPAN border router, it will have a long lifetime.

## 6. Proposed Architecture Realism and limitations

There are several simulation tools for Fog environments including iFogSim and CloudSimSDN [88]. These simulation tools offer different functionalities and features. As such, there is no simulation tool that fulfills all the requirements for every research. For example, iFogSim does not support mobility and uses only tree-based topology to represent Fog nodes [88]. Therefore, in our research, we can not use iFogSim since a feature of our proposed architecture is mobility. Furthermore, our Fog topology is not tree-based.

Nevertheless, a more realistic Fog simulator (other than Contiki/Cooja) can be used that includes characteristics such as low-latency and mobility support. Therefore, such Fog simulator will add value to our proposed architecture.

In this article, we showed how trust modeling can be mapped into our proposed architecture without proposing nor evaluating a trust model. Although the focus of this article is not to propose nor evaluate a trust model, the lack of analysis of a trust model in the proposed architecture can be viewed as a limitation.

## 7. Conclusions and Future Work

In this article, we presented an architecture for modeling trust in IoT environments. Modeling trust in IoT needs to utilize an architecture that is scalable and efficient in term of (a) distributing the load among participating nodes, (b) resolving trust queries, (c) resilience to node failures, and (d) energy consumption.

Our proposed architecture utilizes the Chord protocol and was evaluated through extensive simulation studies under various conditions. Simulation results indicate that our architecture balances the load of storing key values among participating nodes on average. This feature of our architecture improves responsiveness and increases availability.

Another significant advantage of our architecture is its resiliency during churn. That is, the ability of our architecture to stay connected while nodes join or leave the network. Under such conditions, simulation results show that there are no significant lookup failures and thus our architecture is able to function by forwarding/resolving queries and repairing its connectivity.

Scalability of our architecture was investigated by examining the number of nodes traversed to resolve a query. Obtained results indicate with high probability that the typical path length is O(log(N)), where *N* in the number of nodes. Note that on average our proposed architecture resolves queries in 1/2×O(log(N)). Furthermore, lookup latency was evaluated and the evaluation results show that as the number of nodes increase, the lookup latency grows slowly, and again this reinforces the scalability factor of our architecture.

Feasibility of deploying our architecture in resource constrained devices was also examined in terms of energy consumption. It was found that using ContikiMAC as an underline RDC protocol to simulate 64 nodes, the average consumed energy is 9.2 Joules per hour. This indicates even if the Fog node is a 6LoWPAN border router, it will have a long lifetime.

As of future work, we plan to extend our architecture to other IoT layers having multiple Chord rings at the cloud, Fog, and the things layers. Such hybrid architecture will enable efficient and scalable vertical as well as horizontal communication between IoT devices. We are also working on a trust model that is hosted at the Fog layer and utilizes our proposed architecture.

## Figures and Tables

**Table 1:** Simulation configuration.

Parameter	Value
Platform	Contiki v3.0 with Cooja simulator
Network topology	Random
Mote type	Wismote with 16 KB RAM and 128 KB ROM
Network scale	Variable (8, 16, 32, 64, 128 and 256 nodes)
Network layer	RPL + uIPv6 + 6LoWPAN
Radio environment	Unit Disk Graph Medium (UDGM)
Transmission range	60 m
MAC & PHY	Carrier Sense Multiple Access (CSMA) /Collision Avoidance (CA) & IEEE 802.15.4
RDC/ CCR	ContikiMAC/64 Hz and NullRDC/128Hz
Number of iterations	10 iterations

## Data Availability

Not applicable.

## Abbreviations

The following abbreviations are used in this manuscript:

IoS	Internet of Services
CPS	Cyber Physical Systems
FCD	Fog Computing Domain
IoT	Internet of Things
DHT	Distributed Hash Table
